# Protocol: changes in rates of opioid overdose and poisoning events in an integrated health system following the introduction of a formulation of OxyContin® with abuse-deterrent properties

**DOI:** 10.1186/s40360-016-0064-y

**Published:** 2016-05-14

**Authors:** Shannon L. Janoff, Nancy A. Perrin, Paul M. Coplan, Howard D. Chilcoat, Cynthia I. Campbell, Carla A. Green

**Affiliations:** 1 Center for Health Research, Kaiser Permanente Northwest, 3800 N. Interstate Avenue, Portland, OR 97227 USA; 2 Purdue Pharma, L.P., One Stamford Forum, Stamford, CT 06901 USA; 3 Indivior Inc., 10710 Midlothian Turnpike, Suite 302, Richmond, VA 23235 USA; 4 Kaiser Permanente Division of Research, 2000 Broadway, Oakland, CA 94612 USA

**Keywords:** OxyContin®, Opioids, Overdose, Poisoning, Prescription drug monitoring

## Abstract

**Background:**

Addiction, overdoses and deaths resulting from prescription opioids have increased dramatically over the last decade. In response, several manufacturers have developed formulations of opioids with abuse-deterrent properties. For many of these products, the Food and Drug Administration (FDA) recognized the formulation with labeling claims and mandated post-marketing studies to assess the abuse-deterrent effects. In response, we assess differences in rates of opioid-related overdoses and poisonings prior to and following the introduction of a formulation of OxyContin® with abuse-deterrent properties.

**Methods/Design:**

To assess effects of this formulation, electronic medical record (EMR) data from Kaiser Permanente Northwest (KPNW) and Kaiser Permanente Northern California (KPNC) are linked to state death data and compared to chart audits. Overdose and poisoning events will be categorized by intentionality and number of agents involved, including illicit drugs and alcohol. Using 6-month intervals over a 10-year period, trends will be compared in rates of opioid-related overdoses and poisoning events associated with OxyContin® to rates of events associated with other oxycodone and opioid formulations. Qualitative interviews with patients and relatives of deceased patients will be conducted to capture circumstances surrounding events.

**Discussion:**

This study assesses and tracks changes in opioid-related overdoses and poisoning events prior to and following the introduction of OxyContin® with abuse-deterrent properties. Public health significance is high because these medications are designed to reduce abuse-related behaviors that lead to important adverse outcomes, including overdoses and deaths.

**Electronic supplementary material:**

The online version of this article (doi:10.1186/s40360-016-0064-y) contains supplementary material, which is available to authorized users.

## Background

As opioid-related risks to public health have become apparent [1–9] various efforts have been implemented to mitigate opioid-related negative outcomes. States have implemented prescription drug monitoring programs [10, 11], and the FDA now requires manufacturers of long-acting opioids to develop Risk Evaluation and Mitigation Strategies (REMS) and encourages opioid prescribers to complete REMS-compliant education programs [12]. Manufacturers of long-acting opioids have also begun reformulating products to include abuse-deterrent properties, with the goal of reducing prescription opioid abuse [13]. In April 2010, the FDA approved a reformulation of OxyContin® (manufactured by Purdue Pharma L.P.) with abuse-deterrent properties. The goal of the abuse-deterrent formulation was to make it difficult to crush, cut, break or liquefy pills, reducing likelihood that the medication could be snorted, smoked, or injected. The reformulated product was introduced to the market in August 2010. In April 2013, the FDA approved labeling claims that OxyContin® was expected to result in “reduced abuse through intranasal and injecting routes” [14], although abuse by these methods, and the oral route, is still possible [15]. Evidence since its introduction shows that rates of abuse have diminished [16, 17] and then leveled off [15], as have overdoses attributed to prescription opioids [18]. Calls to poison centers related to OxyContin® abuse, accidental exposures, and therapeutic errors were similarly reduced [19, 20]. In contrast, heroin use and heroin overdoses have increased substantially in recent years [18, 21], attributable to the prescription drug epidemic [22] and, in part, to switching from abuse-deterrent opioid formulations to heroin [23]. No harms or adverse effects have been identified related to the physical/chemical properties of the reformulated medication when it is abused, though such harms have been documented in the case of other medications, including injection of temazepam gel capsules [24], and thrombotic thrombocytopenia purpura resulting from injection of abuse-deterrent extended-release Opana [25].

Despite this evidence, a comprehensive assessment of the effects of abuse-deterrent OxyContin® on overdose is lacking. The study protocol described here is a mixed methods research project designed to address that gap by using full electronic medical records of two large integrated health plans, linked to state death data, and to interviews with individuals experiencing overdoses, or their family members. We analyze overdose rates among individuals with active opioid prescriptions, classified in groups, and among individuals without opioid prescriptions. We also examine changes in heroin-related overdoses. Analyses are based on patient electronic medical record (EMR) data, chart audits, and in-depth qualitative interviews.

## Methods

### Study design

This mixed methods study is designed to compare rates of overdose and poisoning events before and after the August 2010 introduction of the abuse-deterrent formulation of OxyContin®. It is one of a series of post-marketing research protocols required by the FDA and funded by Purdue Pharma L.P., the manufacturer of OxyContin®. The primary specific aims of the study are to:

1. Assess the validity of International Classification of Diseases (ICD-9 and ICD-10) diagnoses to accurately identify and categorize opioid-related overdoses and poisonings using chart audits.
2. Estimate rates of, and compare trends in, opioid-related overdoses among all members of the participating health plans:
  - before and after the introduction of OxyContin® with abuse-deterrent properties,
  - among patients with and without active opioid prescriptions,
3. Compare the ratio of rates of opioid-related overdoses and poisoning events among patients prescribed and dispensed OxyContin® with rates in comparator opioid groups, 2 years prior to and 2 years following the introduction of the new formulation of OxyContin®.
4. Conduct exploratory in-depth interviews with a subset of patients who experience opioid-related overdoses and poisoning events (or their relatives) to examine and understand circumstances surrounding overdose events, and involvement of OxyContin®. Triangulate data with chart audit data to describe substances involved in overdoses, including heroin, and assess whether individuals abusing OxyContin® switch to heroin in response to the new formulation.

### Setting

The setting for this study is Kaiser Permanente Northwest (KPNW) and Kaiser Permanente Northern California (KPNC), nonprofit, group model, integrated health systems serving about 3.9 million members (500,000 in KPNW and 3.4 million in KPNC). KPNW and KPNC provide outpatient and inpatient medical, mental health, and addiction treatment services and they maintain integrated EMRs that contain comprehensive administrative and treatment data on all members. Though the majority of the membership in both health plans is comprised of individuals with private insurance, both plans cover substantial numbers of Medicare members and, to a lessor extent, Medicaid members. Consistent with other settings, substance abuse and misuse are common, and the health plans’ membership is generally representative of the populations in the geographic areas they serve. The study protocol and all study procedures are reviewed, approved, and monitored by the Research Subjects Protection Office of the Institutional Review Board at KPNW.

### Population

The study population includes all members of the KPNW and KPNC health systems from February 1, 2003 through July 1, 2013. The total sample size will include approximately 7,500,000 unique individuals across the two health plans (~1,100,000 from KPNW and ~6,400,000 from KPNC).

## Aims 1–3: quantitative data collection

### Opioid-related overdoses and poisoning event identification and categorization

Overdoses and poisonings are identified through ICD-9 and ICD-10 codes in EMRs and state death records, and linked to clinical and administrative data, including pharmacy dispense records (including medication dispensed, dose and days supply), inpatient and outpatient records, and insurance claims for services received outside the health plans (e.g., emergency department visits to non-health plan hospitals). Dispense records are available for nearly all health plan members, and dose and days supply are used to calculate morphine equivalents [26] for each individual included in analyses. Cases are defined as poisonings (Case 1; see Table 1) or overdoses (Case 2; see Table 2) using diagnosis and cause-of-death codes. When multiple events occur for a given person, events on sequential days are considered the same event and events on non-sequential days are considered unique. Death data from Washington, Oregon, and California are incorporated to capture additional opioid-related overdoses and poisoning events that may have resulted in deaths not captured in the KPNW or KPNC EMR systems.

**Table 1:** ICD poisoning codes used to identify overdoses^a^

	ICD 9 code	ICD 10 code
Poisoning by opium (alkaloids) unspecified	965.00	
Poisoning by heroin	965.01	
Poisoning by methadone	965.02	
Poisoning by other opiates and related narcotics	965.09	
Accidental poisoning by heroin	E850.0	
Accidental poisoning by methadone	E850.1	
Accidental poisoning by other opiates and related narcotics	E850.2	
COD: Poisoning by opiates and related narcotics	9650^b^	
COD: Poisoning by opium		T40.0
COD: Poisoning by heroin		T40.1
COD: Poisoning by other opioids		T40.2
COD: Poisoning by methadone		T40.3
COD: Poisoning by other synthetic narcotic		T40.4
COD: Accidental poisoning by and exposure to narcotics and psychodysleptics, not elsewhere classified		X42
COD: Intentional self-poisoning by and exposure to narcotics and psychodysleptics, not elsewhere classified		X62
COD: Undetermined poisoning by and exposure to narcotics and psychodysleptics, not elsewhere classified		Y12

^a^ *Case definition is met when a person has any diagnostic code from the Case 1 list*

^b^ *For the year of 1998 only; COD codes changed to ICD 10 in 1999; ICD 9 codes used for death data during 1998 did not include the fifth digit, which is why this particular code is four digits and without a decimal*

**Table 2:** Opioid adverse effect codes used in combination with related diagnostic codes used to identify overdoses. To meet criteria, a case had to include one diagnosis from category A and one more diagnoses from category B on the same date

Diagnostic category	Diagnosis	ICD 9 code	ICD 10 code	HCPCS code
A	Adverse effects of heroin	E935.0		
A	Adverse effects of methadone	E935.1		
A	Adverse effects of other opioids and related narcotics	E935.2		
A	COD: Adverse effects of opioids and related analgesics		Y45.0	
B	Mixed acid–base balance disorder	276.4		
B	Drug-induced psychotic disorders^a^	292.1		
B	Drug-induced delirium	292.81		
B	Drug-induced mental disorder^b^	292.8		
B	Pneumonia, organism unspecified	486		
B	Chronic airway obstruction, not elsewhere classified	496		
B	Acute respiratory failure	518.81		
B	Other pulmonary insufficiency, not elsewhere classified	518.82		
B	Rhabdomyolysis	728.88		
B	Alteration of consciousness	780.0		
B	Altered mental state	780.97		
B	Apnea	786.03		
B	Shortness of breath	786.05		
B	Dyspnea and respiratory abnormalities—other	786.09		
B	Painful respiration	786.52		
B	Asphyxia and hypoxemia	799.0		
B	Poisoning by opiate antagonists	970.1		
B	Suicide and self-inflicted injury	E950–E959		
B	Assault by drugs and medicinal substances	E962.0		
B	Injection, Naloxone Hydrochloride			J2310

^a^ *Including 292.11 and 292.12*

^b^ *Excluding 292.81*

Opioid-related overdoses and poisoning events are linked to pharmacy dispense records to determine which opioid(s), if any, are active at the time of the event. Since patients on opioid therapy may receive more than one active opioid (e.g., one sustained-release opioid for chronic pain and another short-acting opioid to be used as needed for breakthrough pain), we group and categorize opioids in a hierarchical structure to allow for only one active opioid medication category to be associated with any identified opioid-related overdose or poisoning event. Information about additional involved medications, illicit drugs, and alcohol are also collected. Because we are most interested in trends following the introduction of OxyContin® with abuse-deterrent properties, for comparison purposes, we classify opioid medications using a hierarchy with OxyContin® (including generic single ingredient sustained-release oxycodone) at the top level, followed by single-ingredient immediate release oxycodone, then other class REMS opioids, other opioids (including multiple ingredient immediate release opioids), and no opioids (i.e., no pharmacy record indicating an active opioid prescription at the time of the event). It the latter case, opioid overdoses are considered indications of illicit use. Table 3 details this classification structure and the information related to specific medications included in each of these categories. The study period for capturing trends in opioid-related overdoses and poisoning event rates is February 1, 2003 through July 1, 2013. Trends in opioid-related overdoses and poisoning rates are estimated for each 6-month interval.

**Table 3:** Classification structure for specific medications included in opioid medication hierarchy

Category	Group	Generic name
1	OxyContin®	n/a
	Oxycodone SISR	oxycodone SR
2	Oxycodone SIIR	oxycodone
3	Other Class REMS	fentanyl transdermal patch
		hydromorphone
		methadone
		morphine
		oxymorphone
		opium
		tapentadol
4	Other Opioids (oxycodone MIIR)	oxycodone + acetaminophen
		oxycodone + ibuprofen
		oxycodone + aspirin
	Other Opioids^a^	hydrocodone + acetaminophen
		codeine
		codeine + acetaminophen
5	No Opioid	

^a^ *Includes all codeine and hydrocodone combination products*

#### State death data

State death data (ICD-10 codes for underlying cause of death, contributory cause of death, and immediate cause of death) are transferred to each site, incorporated into the site’s data warehouse (harmonized across sites) using a matching algorithm to identify health plan members, and linked to electronic medical records and administrative data for use in analyses. Only data from health plan members are retained. All death data are available for the full study period for KPNW members, though only underlying cause of death is available for the full study period for the KPNC membership. Therefore, we include only underlying cause of death in primary analyses. It is possible that deaths outside the relevant states may be missed, but believe this to be a negligible problem.

#### Descriptive information

Demographic information available through administrative systems will be collected, including age, gender, race/ethnicity, Medicaid insurance, Medicare insurance. Diagnostic data for the year preceding overdose or poisoning events will also be collected for descriptive purposes (e.g., history of substance use disorders; history of psychiatric disorders).

### Aim 1: data collection for opioid-related overdoses and poisoning event validation

To assess the validity of using EMR diagnoses to accurately identify and categorize opioid-related overdoses and poisoning events, chart audits are conducted with a subset of identified opioid-related overdoses and poisoning events (Aim 1). Audits are conducted for the 2 years prior to the introduction of the reformulated OxyContin® and the 2 years following its introduction (August 1, 2008 through July 31, 2012). Provided with health record numbers, event dates, and inclusion diagnoses, chart auditors review the EMR to locate the identified event for each person and then compile all associated records for that event. Records used may include history and physical records, discharge summary, medication activity report, telephone encounters, and any other related documentation. All printed material and forms are kept in confidential, locked files at all times when not in use.

Staff training begins with a sample of events reviewed by all chart audit staff, adjudicators, and project investigators at both sites to identify any issues with the chart audit form, clarify questions, and ensure consistency in review. A weekly teleconference call with chart audit staff, investigators, expert adjudicators, and administrative staff is held to identify and resolve questions related to the events and the chart audit process. At each site, 200 of the first sample of charts are adjudicated by senior research staff. Once the chart review form and associated instructions are finalized, abstractors work on individualized opioid-related overdoses and poisoning event lists. Biweekly teleconferences are convened for chart audit staff to discuss and resolve individual cases and to refine definitions and instructions as needed.

The chart audit form is completed by auditors to document the causal opioid(s), additional contributing medication(s), contributing alcohol or illicit drug use, prescription detail (dose and frequency), route of administration for each substance, source of each substance when available (e.g., prescription, friend, family member, Internet, street, etc.), and any indication of misuse, abuse, or over-administration for each substance. Auditors also use the form to record administration and response to naloxone hydrochloride and whether the event is solely related to anesthesia administered for a procedure. Events solely related to anesthesia are not abstracted further.

Chart auditors use a study-designed medication codebook to document all substances related to the identified opioid-related overdoses and poisoning event. This codebook lists all opioids in each comparator group, prescription medications verified to have moderate or severe interactions with opioids, and over-the-counter medications known to interact with opioids as well as alcohol and illicit substances. The codebook includes 1355 medications and substances and is used to identify all medications and substances involved or potentially involved in each opioid-related overdose and poisoning event. Following each review, chart auditors summarize the findings as follows:

1. The extent to which substances are involved with the event (only one option is selected)
  - Not an opioid event (no mention of opioids).
  - Single opioid event (only one opiate is involved in the event; no other medications or other substances likely contribute, per documentation).
  - Poly-substance opioid event (at least one substance in addition to an opioid likely or possibly contributes to the event, per documentation).
  - Event unrelated to opioid use (there is a mention of opioids at the time of the event but opioids *did not* contribute to the event).
2. Whether the event appears to be one of the following (only one option is selected):
  - Miscode (diagnostic code or codes that appear to have been applied in error, or documentation of event that does not match the codes applied to that event).
  - Misidentification (diagnostic code or codes and medication are both correct but are not related to each other and not of interest to the study).
  - Neither of the above (documentation in chart is consistent with the EMR-based identification of the event).

When an event is deemed to be miscoded or misidentified, the audit is stopped and the event is sent to an expert adjudicator for confirmation and further documentation regarding the specifics of the miscode or misidentification determination. This information is logged separately from the audit form. These data are compiled and are analyzed separately to look for patterns in inconsistently or incorrectly applied ICD codes within the EMR system.

For all events determined to be consistent with EMR-based identifications as opioid-related overdoses and poisoning or opioid-related adverse effects, data collection staff then determine the type of event (only one option is selected):

- Intentional opioid-related overdose or and poisoning (EMR records are clear that the event was intentional [e.g., suicide or attempt] and involved opioids [single-opioid or poly-drug]).
- Unintentional opioid-related overdoses or poisoning (EMR records are clear that the event was not intentional [e.g., trying to get high; medication error] and involved opioids [single-opioid or poly-drug]).
- Adverse effect related to opioid use (event related o opioid use but did not require intervention beyond adjusting or discontinuing medication for resolution). Sensitivities to properly administered medications are coded here unless they require additional intervention to resolve symptoms.

For events that have no EMR information available on the provided date, outside claims data from both sites are reviewed by senior research staff to provide an indication of intentionality or number of substances involved in the event. These cases are visits and treatments provided at non-KPNW or non-KPNC facilities (e.g., ambulance transport for overdose to the nearest hospital). Determinations made from outside claims data are confirmed with clinical staff and the study PI according to the following criteria:

- Intentional vs unintentional: If coding documents any indication of suicide (e.g., attempt, ideation, self-inflicted) the event is determined to be “intentional”. If there is no such mention, the event is coded “unintentional”.
- Single vs poly-drug: If there is an indication of more than one substance based on outside claims coding (e.g., alcohol intoxication and accidental opiate poisoning) the event is coded “poly-drug”. If only one substance is recorded as poisoning (e.g., methadone poisoning) the event is coded “single”. If only one substance is recorded but additional opiate poisoning or abuse codes are also applied (e.g., heroin poisoning and opioid abuse) the event is coded “single”.
- If there is no information in the claims data related to opioid use, the event is coded as “not an opioid event”.

Each audit file is reviewed for missing data prior to data entry; if forms are incomplete, the file is returned to the staff person who collected the data for completion. Ten percent of charts are reviewed by two reviewers to assess and maintain high inter-rater reliability (>95 %). All identified errors are discussed and corrected. Once abstraction files are complete, data are entered into an electronic database using double entry verification until adequate accuracy is obtained (less than 1 error/100 entries). Once this level is achieved, 10 % of data are double-entered as a continuous check. Following entry, data files from both sites are merged for analysis.

## Aim 4: qualitative data collection

To understand the circumstances surrounding opioid-related overdoses and poisoning events, we conduct in-depth interviews with a subset of patients who experience an opioid-related overdoses and poisoning event. We also conduct interviews with family members of patients who died as a result of an opioid-related overdoses and poisoning event.

Interview candidates (*n* = 90) are identified from the sample of KPNW member with identified opioid-related overdoses or poisoning events. Family members are identified using subscriber account information linked to the decedent’s EMR information, when such information is available.

We sample randomly from purposefully derived pools of people with and without active prescriptions for opioids and oversampling within key subgroups. Pharmacy records are reviewed to determine whether a person with an opioid-related overdose and poisoning event had an active opioid prescription at the time of the event. Because numbers of OxyContin®-associated events are small, we oversample members with active OxyContin® or sustained release oxycodone prescriptions at the time of the event. We also oversample members with no active opioid prescription (no opioid group) in order to identify people engaged in non-medical use of opioids. Members with events in the remaining three comparator groups (oxycodone SIIR, other class REMS, and other opioid) are sampled proportionally based on the number of total opioid-related overdoses and poisoning events identified in each of those categories. We also attempt to balance our sample to obtain roughly equal numbers of male and female participants in each of the opioid categories.

### Recruitment

Potential participants are recruited using mailed letters with follow-up telephone calls inviting participation in a one-time interview and promising compensation of $50. For deceased health plan members whose medical records indicate that opioid overdose was the cause of death and who have no other members associated with their subscriber unit, we mail a letter to the deceased member’s most recent address.

To improve recall regarding the circumstances surrounding opioid-related overdoses and poisoning events, interview candidates are identified from the opioid-related overdose and poisoning event sample from the 6 months prior to the introduction of OxyContin® with abuse-deterrent properties through the years following the introduction. Individuals with more than one active opioid prescription at the time of the opioid-related overdoses and poisoning event (e.g., a sustained-release formulation and an immediate-release formulation for breakthrough pain) are sampled based on their categorization into their “highest” medication comparator group in the hierarchy.

### Interviews

The purpose of the qualitative interviews is to gain further insight into patients’ experiences of overdoses and poisoning events; with family members the goal is to understand as much as possible about the decedent’s experiences prior to overdose. Interviews with patients are semi-structured and focus on pain history, initiation of analgesic medications, switches in prescribed opioid medications or change in dose, misuse of opioids or other prescription medications, illegal drug use and abuse history, the circumstances leading up to and culminating in the specific overdose or poisoning event identified through the EMR, and any post-event treatment plans, medication changes or changes in drug use activity. Because many people experience more than one opioid-related overdose and poisoning event, those additional events may also be explored. We also ask about the opioids individuals were taking, other prescribed medications at the time of the events, contributing alcohol or illicit drug use, prescription details (dose and frequency), route of administration for each substance, and source of each substance (e.g., prescription, friend, family member, Internet, street, etc.). Interviewers also explore indicators of of misuse, abuse, or over-administration for each substance as well as mental health status. Interviews with family members focus on similar questions.

Interviews are conducted using a semi-structured interview guide (see Additional files 1 & 2) to ensure similar questions are asked of all participants. Additional prompts and questions are added during individual interviews to further explore important information.

Experienced master’s- and doctoral-level staff members conduct these hour-long interviews. Participants consent to participate in the interview portion of the study and are provided a copy of their signed consent form and a $50 gift card to a local supermarket chain upon interview completion.

## Data analysis

### Aim 1 analyses: assess the validity of ICD-9 and ICD-10 diagnoses to accurately identify and categorize opioid-related overdoses and poisonings using chart audits

Opioid-related overdoses and poisoning events are identified and then compared to chart audits using the following approach: We use individual and combined ICD-9 and ICD-10 codes from the KP Virtual Data Warehouse (VDW), linked to state death data. The VDW, contains comparable data across multiple participating sites, including KPNW and KPNC, for the conduct of research, including enrollment, demographics, tumor registries, pharmacy dispenses, census data, vital signs, and diagnoses and procedures. We then calculate positive predictive value of EMR-based diagnostic codes compared to chart audit determinations and describe final chart audit determinations and categorizations for each code. Overdoses are also described using chart audit-based categorizations (e.g., suicide event or attempt; polydrug event).

### Aim 2 analyses: estimate rates of, and compare trends in, opioid-related overdoses before and after the introduction of OxyContin® with abuse-deterrent properties, among patients with and without active opioid prescriptions, and those involving heroin

For Aim 2, we compare trends in rates of opioid-related overdoses and poisoning events associated with OxyContin® to rates of opioid-related overdoses and poisoning events associated with other oxycodone and opioid formulations over a 10-year period (February 1, 2003-July 30, 2013). Rates are computed in 6-month intervals from the 7 years prior to the reformulation of OxyContin® and through the 3 years following the introduction of the new formulation. These rates are graphed over time for each category of opioid (immediate-release single ingredient oxycodone, other long-acting opioids for which the FDA has required REMS, and other Schedule II opioids), and well as overdoses among individuals with no active opioid prescriptions to compare trends across all categories. Prior to 2005, all extended-release (ER) oxycodone distributed in the marketplace was branded OxyContin®. In 2005, several generic manufacturers challenged the patent for branded OxyContin® and began to sell generic extended-release oxycodone. The proportion of branded ER oxycodone (OxyContin®) versus generic ER oxycodone declined rapidly. The manufacturer of OxyContin®, Purdue Pharma, L.P., won the patent back in January 2008 and the proportion of branded versus generic ER oxycodone rapidly increased to approximately 85 % of ER oxycodone used in the U.S. To address these changes in the marketplace, the rates of overdose and poisoning in the period prior to the introduction of the new formulation are calculated separately for branded and generic ER oxycodone. If similar, they will be combined and used as the pre-introduction rates, then compared to the post-introduction rates for branded ER oxycodone.

#### Calculation of rates

Poisonings and overdoses related to opioids are the opioid-related events of interest. Dependent variables are the rates of these events in 6-month intervals. We compute rates in two ways. First, we compute the rate of events per number of people with a dispense in each category. For example:

$$ \frac{\mathrm{Number}\ \mathrm{o}\mathrm{f}\ \mathrm{poisonings}/\mathrm{overdoses}\ \mathrm{f}\mathrm{o}\mathrm{r}\ \mathrm{people}\ \mathrm{with}\ \mathrm{a}\ \mathrm{dispense}\ \mathrm{o}\mathrm{f}\ \mathrm{OxyConti}{\mathrm{n}}^{\circledR }/\mathrm{oxycodone}\ \mathrm{S}\mathrm{R}}{\mathrm{Number}\ \mathrm{o}\mathrm{f}\ \mathrm{people}\ \mathrm{with}\ \mathrm{a}\ \mathrm{dispense}\ \mathrm{o}\mathrm{f}\ \mathrm{OxyConti}{\mathrm{n}}^{\circledR }/\mathrm{oxycodone}\ \mathrm{S}\mathrm{R}} $$

Second, we compute the rate per total morphine equivalent in milligrams by category (but including all prescribed opioids). For example:

$$ \frac{\mathrm{Number}\ \mathrm{o}\mathrm{f}\ \mathrm{poisonings}/\mathrm{overdoses}\ \mathrm{f}\mathrm{o}\mathrm{r}\ \mathrm{people}\ \mathrm{with}\ \mathrm{a}\ \mathrm{dispense}\ \mathrm{o}\mathrm{f}\ \mathrm{OxyConti}{\mathrm{n}}^{\circledR }/\mathrm{oxycodone}\ \mathrm{S}\mathrm{R}}{\mathrm{Morphine}\ \mathrm{equivalent}\ \mathrm{milligrams}\ \mathrm{o}\mathrm{f}\ \mathrm{a}\mathrm{ll}\ \mathrm{dispense}\mathrm{s}\ \mathrm{o}\mathrm{f}\ \mathrm{OxyConti}{\mathrm{n}}^{\circledR }/\mathrm{oxycodone}\ \mathrm{S}\mathrm{R}} $$

Rates will be computed for each 6-month period for immediate-release single-ingredient oxycodone, other class REMS opioids, and other Schedule II opioids. These two types of rate calculations are used as dependent variables to assess changes in rates of opioid-related overdoses and poisoning events following introduction of the new formulation and to assess secular trends in rates by comparing to the other opioid groups.

### Aim 3 analyses: compare the ratio of rates of opioid-related overdoses and poisoning events among patients prescribed and dispensed OxyContin® with rates in comparator opioid groups, 2 years prior to and 2 years following the introduction of the new formulation of OxyContin®

For the rate ratio analysis for Aim 3, we examine overdose and poisoning rates calculated by dividing the number of events by the total number of person-years during pre-reformulation and post-reformulation time periods. Negative binomial regression analysis will be used to compare rates between the pre- and post- time periods. Because the same health plan members can appear both in time periods, a generalized estimating equations (GEE) approach is used to account for the correlated nature of the data. Rate ratios analysis examining change from pre- to post- reformulation will be conducted for each opioid category and heroin overdose events, per 10,000 person years. All persons in the health plans will be included in these analyses regardless of whether or not they were currently prescribed an opioid.

#### Statistical power

Initial power estimates are based on detecting a change in the rate of overdose/poisoning events after the new formulation of OxyContin among patients with dispenses of OxyContin, as this is the smallest group to be included in analyses. We estimated the number of patients needed to detect various effect sizes when the rate for the two years prior to the new formulation is compared to the rate for the first year following the new formulation (see Table 4). Pilot data in Kaiser Permanente Northwest indicated that approximately 1 % of patients taking OxyContin experienced an overdose with the old formulation. Data form KPNW and KPNC estimates 6711 patients with active prescriptions of OxyContin in a 6-month window. Table 3 summarizes the number of patients with a dispense of OxyContin needed to detect various effect sizes or changes in the rate of overdose events after the introduction of the new formulation.

**Table 4:** Sample size needed to detect various effect sizes

Reduction in rate of overdose/poisoning events	80 % power	90 % power
25 %	16,617	22,239
50 %	3635	4865
75 %	1378	1845

### Aim 4: qualitative data analysis

Interviews are audio-recorded and transcribed verbatim. Study investigators and interviewers review transcripts weekly throughout data collection to ensure transcript accuracy and appropriate interviewing techniques. Coding schemes for the patient interviews and family member interviews are developed separately. Senior research staff use Atlas.ti software [27] to systematically apply codes to interview transcripts. We complete check coding throughout the process to ensure coder consistency; inconsistencies are discussed and resolved by the team, and code definitions revised as needed. We anticipate a coder consistency of at least 80 %, and will work to resolve discrepancies, rework code definitions, and retrain coders if this is not achieved. Once data are coded, we generate theme reports following review of code-based queries. Themes are compared within codes and across codes, and text selected to illustrate common themes. Contradictory text is also identified and collected for inclusion in reports. Interview data are then linked to EMR and chart abstraction data, triangulated and compared.

## Discussion

This study is designed to assess and track changes in opioid-related overdoses and poisoning events prior to and following the introduction of OxyContin® with abuse-deterrent properties. The main goals of the study are to 1) identify and assess trends in opioid-related overdoses and poisoning events during the full study period, 2) verify and validate opioid-related overdoses and poisoning events using chart audits, and 3) understand, from the patient’s or family member’s perspectives, the circumstances surrounding opioid-related overdoses and poisoning events. Findings from this study will be significant for several reasons: First, we will be able to assess the effects of OxyContin® with abuse-deterrent properties on overdose and poisoning events. Second, the study will produce validated methods of identifying opioid-related overdoses and poisoning events that can be used for public health surveillance. Third, we will have documented first-person accounts of the circumstances surrounding and leading up to opioid-related overdoses and poisoning events, and the effects of abuse-deterrent properties on the behaviors of individuals with such events.

### Strengths and limitations

Strengths of the study included the comprehensive data available through electronic medical records that are linked to health plan administrative and claims data, pharmacy dispenses, and state death records. In addition, chart audits and interviews provide in-depth data that aid understanding of overdose events and circumstances surrounding overdoses. Chart audits will provide an assessment of the validity of using diagnostic codes for identifying overdoses and poisonings. Limitations of the study include that it will be carried out in insured populations, although the health plans’ populations are demographically representative of the populations in the geographic areas they serve and information about overdose in insured populations is also lacking. The health plans are likely, however, to underrepresent individuals in the poorest strata and also those with substance use disorders that would negatively affect ability to obtain or maintain insurance. Death data may be incomplete if individuals do not die in the states of Oregon, Washington, or California, where participating health plans are located. This limitation is expected to be negligible.

### Implications for practice

Opioid abuse, dependence, and overdose ruin peoples’ lives, and societal costs are substantial, including lost productivity, increased healthcare costs, and greater criminal justice involvement and costs [9]. Reducing the likelihood of opioid-related overdoses and poisoning events can help both individuals and overall population health. The results of this study will inform clinicians about the effects of adopting opioid medications with abuse-deterrent properties for chronic pain treatment.

## Additional files

Additional file 1: SOURCE Patient Interview Guide. (PDF 27 kb)

Additional file 2: SOURCE Family Member Interview Guide. (PDF 24 kb)

## Abbreviations

ER: extended-release
EMR: electronic medical record
FDA: Food and Drug Administration
KPNC: Kaiser Permanente Northern California
KPNW: Kaiser Permanente Northwest
REMS: Risk Evaluation and Mitigation Strategy
