# Supplementary material for: Protocol: changes in rates of opioid overdose and poisoning events in an integrated health system following the introduction of a formulation of OxyContin® with abuse-deterrent properties
Source: BMC Pharmacol Toxicol. 2016 May 14;17:21. doi: 10.1186/s40360-016-0064-y (PMC4867981; doi:10.1186/s40360-016-0064-y)
Supplement: Supplementary file 1 — SOURCE Patient Interview Guide. (PDF 27 kb) [file 40360_2016_64_MOESM1_ESM.pdf]

SOURCE Patient Interview Guide

I want to start by telling you a little about this interview. First, I'll want to ask you a few questions about who you are and your health, then I want to ask you about the ______ (opioid-related event) you experienced on _______(date). Do you have any questions for me before we start? OK—let's get going…

Section 1 Purpose: To get to know participant and build rapport, and identify key demographic variables.

1. I'd like to start by asking you to tell me a little about yourself...

How long (have you been/were you) a KP member?
Do you work or go to school?
Are you married or in a relationship?
Do you have children?
How is your health?
  Can you tell me about any chronic pain or other chronic conditions you have?
    What about current medications for pain?

2. **I want to ask you some questions regarding the ____________(opioid-related event) that occurred on ____(date) but first I would like to ask a few questions about what was going in your life at that time.** *(Use answers from question number 1 to inform these questions)*

Probes:
Were you working at the same place? Going to the same school?
Were you in your current relationship at that time?
How were things going for you at work/home/school or in your family/social life at that time (*ask about any areas where the person had responsibilities or about things that took up much or their time*)?
How was your physical health at that time?
  Was it similar to your current health/worse/better?
What about your emotional or mental health?
  Was it similar to your current health?
Did you have any significant changes in your life in the year prior to the event (e.g., death of friend or family, changing jobs/schools, divorce, moving, accident/injury, etc)?

Section 2 Purpose: To identify the opioid-related event and understand the details of what happened.

3. Now I'd like to have you tell me a little about the ________ (opioid-related event) that occurred on ________(date). Basically, I'd like to know the details of what happened to you…

Probes:
If accident or crash, probe for details of how opioid was involved.
Any previous history of opioid use before the event (prescription, recreational, both?)?
What opioid(s) was/were taken?
How was opioid taken (route of administration—pill, injected, snorted etc.)?
What symptoms were experienced?
Result in ER visit or hospitalized?
Who else involved and how?
Was overdose intentional?
Were multiple drugs involved (probe for which)?
How were opioids (& other involved drugs as applicable) obtained?
  Prompt for: in the hospital; by prescription from a single doctor; through prescriptions from multiple doctors or providers; given by a friend of relative; taken/stolen from someone else; purchased from a friend, relative, or acquaintance (not a dealer); purchased from a dealer (not a legal pharmacy or provider); purchased from an on-line pharmacy; from multiple sources etc.

4. **Do you remember how much (*opioid*) you were taking/using at the time (*event*) occurred?**

    How often did you take that amount?
    How long had been using that amount?

5. **Were you taking any other medications or drugs around the time of the event?**

Probes
- Were you taking any other medications for pain (prescription, non-prescription, including medical marijuana)?
- What about medications for other physical health conditions?
- Were you taking any medications for mental health reasons (e.g. anxiety, sleep, or depression)?
- Were you drinking alcohol in the time period surrounding the event?
    - How much?
    - How often?
- What about any illegal drugs (e.g. stimulants, other opioides)?
    - How were you getting them?

6. **Can you tell me about any friends or family members that were concerned with your use of medication/substances prior to the event?**

    What were they worried about?
    Did they express their concern to you?
        If they did, what happened or how did they express concern?
        How did you respond to their concern?

7. **Prior to the event, did your healthcare provider recommend anything like:**

    Limiting your use of prescription opioids/ take less/ wean off
    Termination of care
    Prescription contract/ only receive opioids from one provider
    Addiction treatment/ other forms of help
    Changing to other medications or methods

8. **What about following the event—did your clinician recommend anything, like:?**

    Limiting your use of prescription opioids/ take less/ wean off
    Termination of care
    Prescription contract/ only receive opioids from one provider
    Addiction treatment/ other forms of help
    Changing to other medications or methods

9. **What kind of medical care did you receive for the (opioid related event), if any?**

10. **Were you referred to any type of substance treatment program as a direct result of (*event*)?**

11. **If you continued to use (substance[s]) can you describe any changes in your use of (*substance[s]*) following (*event*)?**

12. Can you tell me about any other opioid-related overdoses/accidents/injuries (use participant's term) you've experienced either before this one or since this one?

Probes: If accident, injury or crash, probe for details of how opioid was involved.
Any previous history of opioid use before the event (prescription, recreational, both?)?
How was opioid taken?
What symptoms were experienced?
Result in ER visit or hospitalized?
Who else involved and how?
Was overdose intentional?
Were multiple drugs involved (probe for which)?
How were opioids (& other involved drugs as applicable) obtained?

Prompt for: in the hospital; by prescription from a single doctor; through prescriptions from multiple doctors or providers; given by a friend of relative; taken/stolen from someone else; purchased from a friend, relative, or acquaintance (not a dealer); purchased from a dealer (not a legal pharmacy or provider); purchased from an on-line pharmacy; from multiple sources etc.

13. **When you look back on the event now, (*refer to length of time since the event*) how do you feel about what happened?**

Section 3 Purpose: To get a sense of what has been going on with participant since OOP event.

14. What has been happening since (*event*)?

- **Are you still taking/using (*substance*)?**
- **Are you taking/using other opiates?**
- **Have you gone through any type of formal treatment for your use of (*substance*)?**
- **How do you feel about your (*either current or former*) use of (*substance*)?**
- **Are your medications being managed or monitored differently?**

Section 4 Purpose: To bring the interview to a close, on a positive note.

15. We're just about finished with today's interview. I'm wondering if you have any advice you'd want to give to other people who might be at risk of having a similar kind of event to what you had?

16. Is there anything else you think I should know about this event that I haven't asked about?

17. **Do you have any questions for me at this point?**

18. Thank participant for making an important contribution and transition from interview.
