# Supplementary material for: Protocol: changes in rates of opioid overdose and poisoning events in an integrated health system following the introduction of a formulation of OxyContin® with abuse-deterrent properties
Source: BMC Pharmacol Toxicol. 2016 May 14;17:21. doi: 10.1186/s40360-016-0064-y (PMC4867981; doi:10.1186/s40360-016-0064-y)
Supplement: Supplementary file 2 — SOURCE Family Member Interview Guide. (PDF 24 kb) [file 40360_2016_64_MOESM2_ESM.pdf]

SOURCE Family Member Interview Guide

I want to start by telling you a little about this interview. We're trying to understand what happened to [NAME of relative] so that we can try to prevent similar events from happening to other people. For that reason, I'll ask you a little about [NAME of relative]'s health history and life circumstances. Then I want to ask you about the (opioid-related event) [NAME] experienced when s/he passed away. Do you have any questions for me before we start? OK—please feel free to stop me at any point if you need a break or if you have questions for me…

Section 1 Purpose: To understand any relevant background information.

1. I'd like to start by asking you to tell me a little about [NAME]'s background...

a) How was his/her physical health?
   Prompts: Can you tell me about any chronic pain or other chronic conditions he/she had?
   What about medications he/she was using for pain?

b) How was his/her mental health?
   Prompt: Can you tell me about any mental health diagnoses s/he had?

c) What about any problems with substances or problems with addiction?
   Prompt: Can you tell me about treatment s/he received for substance problems, if any?

2. **Now I'd like to ask you some questions about what was going on in [Relative's name] life in the period before s/he passed away. Can you tell me about anything you think was happening that put him/her at risk for an overdose?**

Prompts: Changes in life situation (death of someone close, loss of job, divorce etc.)?
Change in physical health?
Change in mental health?
Change in problems with substances?

Section 2 Purpose: To understand opioid-related event and relevant details.

3. Now I'd like to have you tell me a little about the event that lead to [Name's] death. Could you tell me what happened, to the best of your knowledge?...

Probes: If accident or crash, probe for details of how opioid was involved.
(*if unknown*) Previous history of opioid use before event (prescription, recreational, both?)?

Questions:

a) What opioids did s/he take?
b) How did [NAME] take the opioid (pill, injected, snorted etc.)?
c) Can you tell me about any other drugs, or alcohol, that s/he took with the opioids?
d) Did [NAME] go to the emergency room or hospital?
e) Was anyone else involved in the event, and how?
f) Do you have any idea whether the overdose was intentional or not?
g) Do you know anything about how [NAME] got the opioids (& any other involved drugs)?
   Prompts: -in the hospital;
   -prescription from a single doctor;
   -prescriptions from multiple doctors or providers;
   -given by a friend or relative;
   -taken/stolen from someone else;
   -purchased from a friend, relative, or acquaintance (not a dealer);
   -purchased from a dealer (not a legal pharmacy or provider);
   -purchased from an on-line pharmacy;
   -from multiple sources etc.

4. **Do you have any idea how much (*opioid*) he/she was taking/using at the time of his/her death? If so, how much?**

   a) How often did he/she take that amount?
   b) How long had he/she been using that amount?

5. **Was [NAME] taking any other medications or drugs around the time of the event?**

   Probes: Was he/she taking any other medications for pain (prescription, non-prescription, including medical marijuana)?
   What about medications for other physical health conditions?
   Was he/she taking any medications for mental health reasons (e.g. anxiety, sleep, or depression)?

6. **Were you or any other friends or family members concerned with [NAME's] use of medication/substances prior to the event?**

   If yes,
   a) What were you/they worried about?
   b) Did you/they express their concern to [NAME]?
      Probes: If so, what happened or how did you/they express concern?
      How did he/she respond to your/their concern?

7. **Do you know if prior to the event, any of [NAME's] healthcare providers ever recommend anything like:**
   a) Limiting his/her use of prescription opioids/ take less/ wean off
   b) Termination of opioid prescriptions
   c) Prescription contract/only receive opioids from one provider
   d) Addiction treatment/other forms of help
   e) Changing to other medications or methods

8. **What kind of medical care did [NAME] receive for the (opioid related event), if any?**

9. **Can you tell me about any other opioid-related overdoses, accidents, or injuries [NAME] experienced before this one?**

   Probes: If accident, injury or crash, probe for details of how opioid was involved.
   How was opioid taken?
   Was the overdose intentional?
   Were multiple drugs involved (probe for which)?

10. **When you look back on the [NAME's death], can you tell me about anything (more) you think the medical care system could have done to prevent this from happening?**

Section 4 Purpose: To bring the interview to a close, on a positive note.

11. **We're just about finished with today's interview. I'm wondering if you have any advice you'd want to give to other people or families who might be at risk of having a similar kind thing happen to them?**

12. **Is there anything else you think I should know about what happened that I haven't asked you about?**

13. **Do you have any questions for me at this point?**

Thank participant for making an important contribution and transition from interview.
